# Bridging the gap: A program case study of the Regional Telemedicine Center of an apex medical center in Southwestern Mindanao, Philippines

**DOI:** 10.1093/oodh/oqaf015

**Published:** 2025-07-09

**Authors:** Jaime Kristoffer Punzalan, Mary Germeyn Punzalan, Jessa Mayet Sumatra-Mabalot, Tristan Jay Amit, Louie Virgil Gallardo, Marichelle Valeros, Afdal Kunting

**Affiliations:** Zamboanga City Medical Center, Dr Evangelista St., Brgy Sta Catalina, Zamboanga City 7000, Philippines; School of Medicine, Ateneo de Zamboanga University, La Purisima St., Zamboanga City 7000, Philippines; Medical informatics Unit, University of the Philippines – Manila, Padre Faura St, Ermita, Manila, 1000 Metro Manila, Philippines; Zamboanga City Medical Center, Dr Evangelista St., Brgy Sta Catalina, Zamboanga City 7000, Philippines; School of Medicine, Ateneo de Zamboanga University, La Purisima St., Zamboanga City 7000, Philippines; Zamboanga City Medical Center, Dr Evangelista St., Brgy Sta Catalina, Zamboanga City 7000, Philippines; Universidad de Zamboanga, Don Toribio Street, Tetuan, Zamboanga City 7000, Philippines; Zamboanga City Medical Center, Dr Evangelista St., Brgy Sta Catalina, Zamboanga City 7000, Philippines; Zamboanga City Medical Center, Dr Evangelista St., Brgy Sta Catalina, Zamboanga City 7000, Philippines; Zamboanga City Medical Center, Dr Evangelista St., Brgy Sta Catalina, Zamboanga City 7000, Philippines; School of Medicine, Ateneo de Zamboanga University, La Purisima St., Zamboanga City 7000, Philippines; Zamboanga City Medical Center, Dr Evangelista St., Brgy Sta Catalina, Zamboanga City 7000, Philippines; School of Medicine, Ateneo de Zamboanga University, La Purisima St., Zamboanga City 7000, Philippines

**Keywords:** telemedicine, specialty care, health workforce, health systems and policy, UTAUT2

## Abstract

Telemedicine is increasingly recognized as a strategic solution for enhancing healthcare access in isolated areas like the Zamboanga Peninsula, Philippines. This study examines the implementation and factors affecting the adoption and effectiveness of the Zamboanga City Medical Center Regional Telemedicine Center, which facilitates specialist care and real-time provider collaboration. Despite its benefits in reducing travel and enhancing patient outcomes, adoption is hindered by challenges such as unreliable network connectivity and complex user interfaces. Key contextual factors—technical, organizational, ethical, financial, political, legal, and socioeconomic—necessitate comprehensive policy improvements, training, and infrastructure upgrades. The study concludes that the success of telemedicine depends on comprehensive support systems and standardized practices to ensure consistent quality and advance regional health coverage.

## INTRODUCTION

Healthcare access is a universal necessity, yet it remains inaccessible to many, especially in regions characterized by significant geographical and socioeconomic disparities [1, 2]. The Zamboanga Peninsula in the Philippines exemplifies this challenge, where the distribution of medical professionals is heavily skewed towards urban centers. This urban concentration results in significant healthcare access disparities, with specialist medical practitioners primarily based in major cities, such as Zamboanga City, Pagadian City, and Dipolog City, and generalist practitioners spread thinly across rural areas [3, 4].

The inequity in healthcare distribution has been further exacerbated by the COVID-19 pandemic, which introduced additional barriers to patient mobility due to stringent border restrictions. This situation highlighted the urgent need for innovative healthcare delivery methods. Telemedicine has responded to this call by leveraging digital technology to bridge the geographic gaps that hinder access to essential healthcare services.

In 2021, recognizing the acute need for integrated healthcare services, a tertiary medical center in Southwestern Mindanao established its regional telemedicine center (RTC). This initiative was not part of a pre-pandemic plan, but rather a direct institutional response to the barriers exposed during the height of the COVID-19 crisis in 2020, particularly the movement restrictions and care disruptions experienced by residents of geographically isolated and disadvantaged areas [5]. The pandemic underscored the urgency of adopting digital health innovations to maintain continuity of care across the region. Planning for the telemedicine center began in the latter half of 2020, culminating in its operational launch in early 2021. The center was designed to connect generalist physicians in remote facilities with specialists at the medical center, thereby enabling timely, expert-guided care that had previously been inaccessible.

As of 2024, the RTC has facilitated patient referrals across a wide range of medical conditions using a custom-built web-based application. The services delivered through the platform encompass both general and specialty care. General health services encompass the remote management of common clinical concerns. These services are typically initiated by general practitioners or municipal health officers in rural facilities who require timely guidance or validation for patient management. The RTC also supports a portfolio of specialty care services, coordinated through scheduled teleconsultation sessions and asynchronous referrals.

This program case study aims to describe the implementation of the RTC, established by a tertiary medical center in Southwestern Mindanao, including its service delivery mechanisms, as well as the personnel, organizational, infrastructural, and sociopolitical environment in which it operates. By examining the individual and systemic factors influencing the adoption of telemedicine, this study aims to provide an evidence-based assessment of how digital health innovations can bridge healthcare access disparities in geographically isolated and underserved regions.

## MATERIALS AND METHODS

### Study design

The program case study employed a multi-level, mixed-methods design to describe the implementation of the RTC and explore the individual and contextual factors influencing the utilization of a medical center’s Telemedicine Center in the Zamboanga Peninsula [6]. A descriptive case study approach was used, integrating document review and systems analysis to capture the program’s development, infrastructure, workflows, and governance mechanisms. A cross-sectional, quantitative design was employed to identify the individual factors influencing the use of telemedicine technology by personnel from various healthcare facilities, utilizing the Unified Theory of Acceptance and Use of Technology (UTAUT) 2 [7, 8]. A descriptive qualitative study, following the consolidated criteria for reporting qualitative studies guidelines, was conducted to explore the contextual factors that affect the use of the Telemedicine Center [9]. The contextual factors encompass technical, organizational, ethical, financial, political, legal, and socioeconomic aspects, providing a comprehensive context for the telemedicine structure [10].

The scope of this study focuses on the healthcare professionals as the primary end-users of the telemedicine platform. While patients are the ultimate beneficiaries of the care provided through the system, they do not directly interact with the telemedicine web application used for referrals and specialist consultations. As such, the study focuses on the perspectives, usage patterns, and contextual experiences of healthcare personnel who operate the system.

This study was conducted following the ethical guidelines and principles. Ethical clearance was obtained from the Ethics Review Board of Zamboanga City Medical Center, with approval reference number 2023–33. Informed consent was obtained from all participants before their inclusion in the study.

### Study population and setting

This study was conducted between October 2023 and January 2024 in various healthcare facilities, including hospitals, infirmaries, and public health units in the Zamboanga Peninsula and its nearby island provinces. These facilities vary in proximity to the apex tertiary medical center, ranging from as close as 30 minutes to as far as 12 hours by land travel and up to 24 hours by inter-island boat travel. The inclusion of both inland and island-based facilities reflects the geographically fragmented nature of the service area and the logistical challenges of providing timely specialist care in these contexts. All partner institutions with an active Memorandum of Agreement with the Apex Medical Center were invited to participate, regardless of the volume or frequency of telemedicine referrals they received. No site was excluded based on prior engagement levels. Facilities were contacted through both remote coordination and personal visits over three months. The final sample comprised those institutions whose healthcare personnel consented to participate and provided complete responses to the survey and/or interviews.

Due to the dynamic nature of staffing and the high turnover of healthcare professionals in rural and remote facilities, it was not feasible to establish a stable sampling frame for this study. As a result, convenience sampling was employed, selecting available and willing participants from the identified partner facilities. While this limits the ability to generalize findings to the entire healthcare workforce involved in the Telemedicine Center, efforts were made to include participants from geographically and institutionally diverse sites, thereby enhancing contextual relevance and capturing operational heterogeneity.

For the qualitative aspect, participants were selected based on their level of involvement with the Telemedicine Center, knowledge of implementation processes, and familiarity with institutional and operational constraints. This approach was deemed sufficient to achieve thematic saturation and provide a well-rounded understanding of systemic facilitators and barriers to telemedicine utilization.

### Data collection

To describe the implementation of the RTC, the study team conducted a structured analysis of the document and system. Primary data sources included internal project documentation such as memoranda of agreement, implementation manuals, user guides, administrative workflows, and training materials. Technical system specifications, backend architecture documentation, and feature lists of the custom-built web application were also reviewed.

Additionally, a functional walkthrough of the telemedicine system was conducted in consultation with the technical development team to map key processes and user flows, including facility onboarding, case referral, triaging, messaging, and specialist consultation. These processes were triangulated with observational notes and system usage statistics (e.g. the number of referrals and types of specialties involved) to validate implementation milestones and identify core components and bottlenecks.

To assess individual factors affecting telemedicine use, this study incorporated survey questionnaires developed by Ly et al. and Shi et al [7, 10]. Ly et al.’s questionnaire gathered data on participants’ sociodemographic characteristics and their general attitudes toward telemedicine utilization [10]. On the other hand, Shi et al.’s questionnaire is a detailed instrument based on the UTAUT 2, a model commonly used to predict the behavioral intentions of individuals when adopting new technologies, such as telemedicine [7]. This questionnaire includes a 23-item scale using a 7-point Likert scale, covering various subscales: performance expectancy, effort expectancy, social influence, facilitating conditions, hedonic motivation, price value, and behavioral intention, each comprising 3–4 items.

Performance expectancy evaluates whether using the technology will enhance job performance. Effort expectancy measures the ease of use of the technology. Social influence refers to the perceived pressure from others to adopt a technology. Facilitating conditions examine the support structure for implementing the technology, including organizational and technical resources. Hedonic motivation looks at the enjoyment of using the technology, while price value considers the cost-effectiveness from the user’s perspective. Finally, behavioral intention gauges healthcare personnel’s willingness to use telemedicine in their practice. Notably, the instrument demonstrated high reliability, with Cronbach’s alpha values for these subscales ranging from 0.890 to 0.957 [7]. This comprehensive framework does not reflect the acceptance of a specific telemedicine center or platform but rather provides a general view of user acceptance of telemedicine, typically facilitated through standard communication methods, such as voice calls, SMS, or messaging applications.

The interview guide was adapted from Ly et al.’s study of contextual factors. Contextual factors play a crucial role in determining the success of telemedicine services. This study examines the technical, organizational, ethical, political, legal, and socioeconomic factors that provide a comprehensive context for the telemedicine structure [10]. Technical factors encompass the operational aspects of telemedicine, including the quality of internet connections, the frequency of technical failures, and the adequacy of training provided to users. Organizational factors relate to the structure and functioning of healthcare institutions, encompassing elements like workload management, healthcare system practices, and the capacity to adapt to internal and external changes. Ethical concerns in telemedicine center on the ethical implications of utilizing this technology, including data security issues and the lack of comprehensive ethical frameworks. Political concerns involve the engagement and support of political actors, the influence of government policies and legislative actions, and the need for standardization in the implementation of telemedicine. Legal influences encompass the liabilities associated with telemedicine services, including civil, criminal, disciplinary, and penal aspects. Finally, socioeconomic factors encompass the cultural and religious beliefs that influence the acceptance and utilization of telemedicine, as well as the potential social conflicts that may arise from its implementation. The interview guide for contextual factors consists of six questions, one for each. Follow-up questions will be asked during the interview as needed.

The informed consent and survey questionnaire were made available in both paper-based and electronic formats via Google Forms. Informed consent was gathered before the respondents could access and answer the survey questionnaire. Once consent was obtained, the respondents completed the UTAUT 2 questionnaire.

After completing the survey questionnaire, consent was requested for an interview. They were contacted to schedule an interview if they agreed to participate. Two [2] interviewers, also authors of this study, conducted the interview. They have several research publications employing a qualitative methodology. Interviewees may be acquainted with the interviewers due to their shared alma mater. The interview guide, adapted from the study by Ly et al., was utilized [10]. The interviews were voice-recorded, transcribed, and translated into English. Once translated, a transcription copy was made available to the participant for feedback.

### Data analysis

The implementation of the RTC was analysed using a descriptive case study approach. Documents were examined for key components of program design: governance structure, clinical workflows, system architecture, and digital infrastructure. System components were organized using a logic model framework that reflected inputs (e.g. infrastructure and personnel), processes (e.g. referral pathways and triaging), and outputs (e.g. referral volumes and services delivered). Observational insights and system usage data were used to validate and enrich the interpretation of implementation fidelity and adaptability.

Quantitative data were analyzed using descriptive and inferential statistics to explore the distribution and variation of responses across the seven subscales of the UTAUT 2. Each subscale was operationalized based on validated constructs: ‘performance expectancy’ measured perceived enhancement of job performance through telemedicine use; ‘effort expectancy’ captured perceived ease of learning and using the platform; ‘social influence’ assessed perceived peer and institutional expectations; ‘facilitating conditions’ evaluated the adequacy of technical and organizational support; ‘hedonic motivation’ measured the degree of satisfaction and enjoyment in using the system; ‘price value’ assessed perceived cost–benefit ratio; and ‘behavioral intention’ captured respondents’ willingness to continue using telemedicine. Each construct was represented by 3–4 items on a 7-point Likert scale, with Cronbach’s alpha coefficients used to assess internal consistency.

Qualitative data from focus group discussions and key informant interviews were analysed thematically. The analysis followed a deductive approach, guided by predefined contextual domains adapted from Ly et al [10]. Audio-recorded interviews were analysed using a framework analysis approach. Initial coding and thematic analysis were conducted independently by two study authors who also served as interviewers. To minimize bias, the preliminary coding framework and emergent themes were subsequently reviewed by two external academic colleagues from separate institutions with expertise in qualitative research and reflexivity. These external reviewers provided critical feedback on theme refinement, categorization, and interpretation. Their suggested revisions were incorporated into the final thematic analysis.

## RESULTS

### Regional telemedicine center

**Figure 1:**
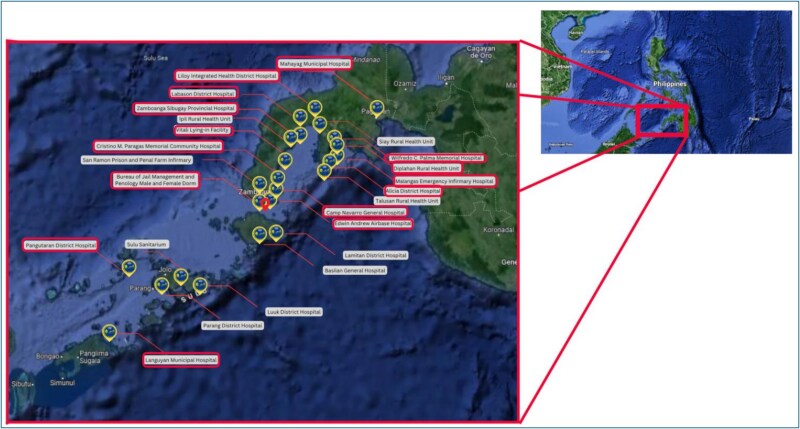
Partner institutions of the RTC. The red pin on the map shows the position of the Apex Medical Center on the Zamboanga peninsula.

The RTC, established in 2021 by a tertiary medical center in Southwestern Mindanao, was designed to address structural inequities in access to specialist care in geographically isolated and disadvantaged areas (see Fig. 1). By June 2024, the RTC had supported 1793 patient referrals facilitated through a secure, in-house web-based platform. This platform enables remote collaboration between generalist providers and specialists using structured case registration, asynchronous messaging, follow-up coordination, and clinical documentation features. It supports a full range of system functions, including physician and patient record management, case tracking dashboards, and communication through text-based notes and the upload of clinical images.

Figure 1 illustrates the geographic distribution of telemedicine partner facilities across the Zamboanga Peninsula and surrounding island provinces. Each pin represents an institution, with annotations indicating the type of facility and the range of specialties accessed. Each red box represents the institutions that were able to participate in either the quantitative or qualitative aspect of this study. Facilities vary in both distance and accessibility from the medical center, demonstrating the program’s regional reach and its ability to overcome spatial health inequities through digital infrastructure.

#### Governance structures and clinical workflows

The ZCMC RTC incorporates several key features to deliver efficient healthcare, particularly in remote areas. The program’s process flow is structured into two main phases: Facility Registration and Patient Referral. Facility Registration involves executing an approved legal agreement between the medical center and the referring facility, preparing hardware requirements, registering the referring facility and its personnel in the web application, and training healthcare personnel to utilize the web application for referral and management purposes. The patient referral phase involves patient registration by the referring healthcare personnel, reception and triage of the patient by the RTC navigator, and case management, where specific treatment needs and specialist guidance are detailed through a short messaging thread feature.

The program’s essential aspect is specialist care access, which significantly expands the availability of specialized medical treatments to patients in rural and remote areas in the Zamboanga Peninsula. The program connects a broad network of specialists to general practitioners and other healthcare personnel through seamless referrals, utilizing a messaging feature that enables the application to transmit short messages and images.

Specialist consultations were provided across 10 major service areas: Internal Medicine, Pediatrics, Obstetrics and Gynecology, General Surgery, Family and Community Medicine, Psychiatry, Dermatology, Ophthalmology, Infectious Diseases, and Rehabilitation Medicine. These departments contributed to a pool of ~10 specialists actively engaged in the program. Patients who benefited from these services include individuals requiring maternal and child health support, chronic disease management, dermatologic assessments, surgical follow-up, and mental health interventions, services that would otherwise require interprovincial travel or go unmet entirely.

During a typical referral, referring providers input patient demographic data, vital signs, presenting complaints, and clinical history into the platform. The system also enables the transmission of diagnostic images, such as wound photographs, skin lesion documentation, ultrasound printouts, electrocardiograph (ECG) tracings, and handwritten prescriptions or laboratory results captured using mobile cameras.

Furthermore, real-time collaboration is a cornerstone of the program. It enables instant communication and collaborative decision-making through features like image transmission and the provision of patient details, including demographics and vital signs. This facilitates a quicker and more accurate diagnosis, enabling a collaborative approach to patient care that ensures all healthcare providers are synchronized and informed at every stage of the treatment process.

#### System architecture and digital infrastructure

The system is designed for ‘provider-to-provider communication only’. All registered users are licensed healthcare professionals, including physicians, nurses, and program navigators. Patients are not granted direct access to the platform and do not interact with the interface. Instead, all patient information is mediated through authorized clinicians, who input demographic data, clinical findings, and diagnostic materials. The platform’s core functionalities include account registration for healthcare providers, case submission, referral triaging, asynchronous messaging, diagnostic image upload, patient record management, follow-up documentation, and referral closure. A front-end dashboard allows clinicians to monitor their submitted cases and track responses from specialists. Communication is primarily text-based but supports the upload of supplemental clinical images such as wound photos, dermatologic lesions, ultrasound printouts, ECG strips, handwritten notes, or lab results—captured via mobile phone or desktop camera. This role-based architecture ensures streamlined clinical decision-making while maintaining professional oversight and documentation integrity across distributed sites.

**Figure 2:**
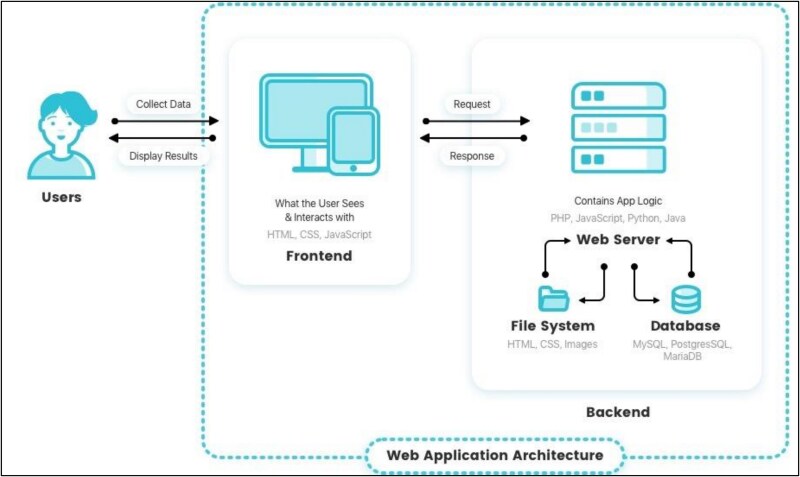
Web application system architecture of the RTC.

The ZCMC RTC leverages a system architecture that enhances system manageability, scalability, and security. The client interface is developed on the front end, supporting the creation of dynamic and interactive user interfaces through its component-based architecture, which allows for efficient updates and rendering (see Fig. 2). This is complemented by a competent library that ensures the user interface is accessible, visually appealing, and consistent, with customizable design components tailored to the needs of healthcare providers. The front end communicates with the back end through an API for efficient data fetching, submission, and real-time updates (See Fig. 2).

To enhance the program’s effectiveness, six [6] facilities have been equipped with desktop computers, while three [3] have been provided with satellite internet access to improve connectivity. This infrastructure investment ensures that even the most remote locations can participate effectively in the telemedicine program, significantly enhancing the accessibility and quality of healthcare services.

The backend is powered by a web application framework designed for server-side applications that handle sensitive medical data. It manages all the business logic, user authentication, and data processing, serving as a conduit between the front end and the database. The framework’s strong security features safeguard against common web threats, enhancing data protection within telemedicine.

A relational database management system stores essential data such as user profiles, patient records, and medical histories in a database. An object-relational mapper facilitates expressive and straightforward interactions with the database, ensuring data integrity and security through features such as transactional support and rollbacks.

Additional system components include a backend dependency management and a runtime environment for managing JavaScript packages on the front end, streamlining the integration of third-party libraries. The entire application is hosted on a cloud hosting provider that ensures high availability and performance. Additionally, pipelines automate the testing and deployment processes, allowing seamless, downtime-free updates.

### Healthcare personnel context

**Figure 3:**
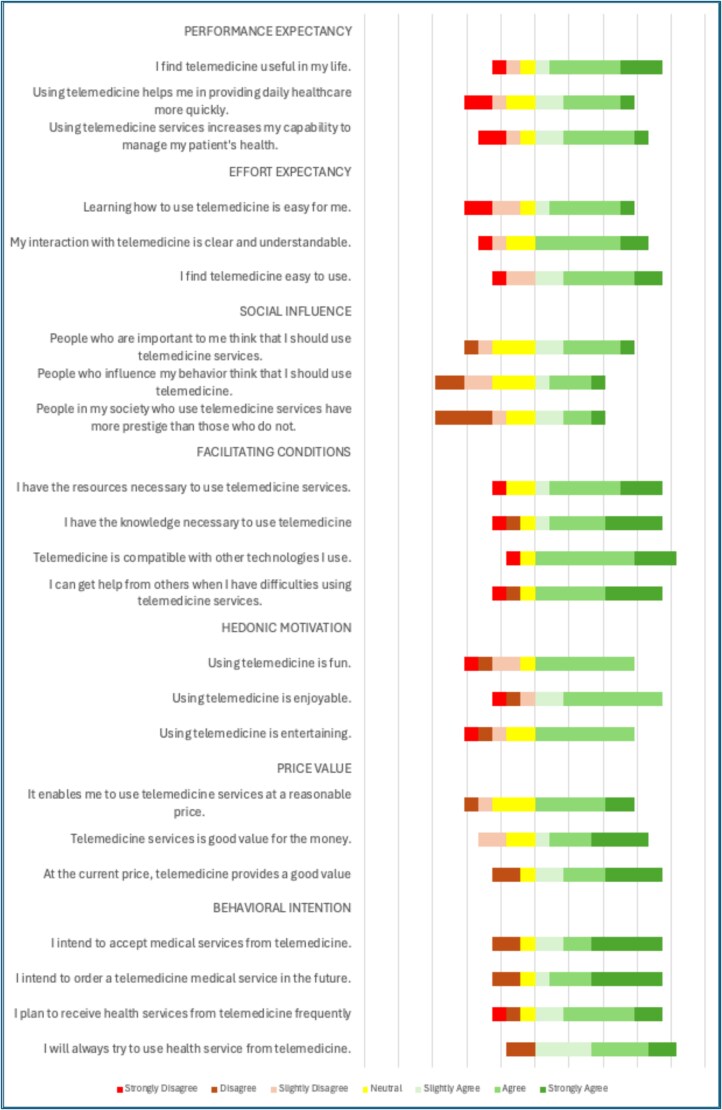
Proportions of healthcare personnel on the 7-point Likert scale based on the unified theory of acceptance and utilization for technology (UTAUT 2) for telemedicine

A survey using the UTAUT 2 framework was conducted to assess the perceptions of healthcare personnel on telemedicine across various facilities (See Fig. 3). A total of 15 healthcare personnel participated in the pilot survey, which evaluated telemedicine adoption using the UTAUT2 framework. These respondents were drawn from 13 participating healthcare facilities across the Zamboanga Peninsula and represented frontline users of the RTC.

Of the ‘15 healthcare personnel’ who participated in the quantitative survey, ‘60% (*n* = 9)’ were female and ‘40% (*n* = 6)’ were male. The majority of respondents ‘(53.3%, *n* = 8)’ were between ‘25 and 35 years old’, followed by ‘26.7% (*n* = 4)’ aged ‘36 to 45’, and ‘20% (*n* = 3)’ over ‘45 years old’. In terms of professional experience, 46.7% (*n* = 7) had **<**5 years of clinical experience, ‘33.3% (*n* = 5)’ had between ‘5 and 10 years,’ and ‘20% (*n* = 3)’ had ‘more than 10 years’ of experience. Regarding healthcare roles, ‘46.7% (*n* = 7)’ were physicians, ‘33.3% (*n* = 5)’ were nurses, and the remaining ‘20% (*n* = 3)’ held allied or administrative roles that directly supported the implementation and coordination of telemedicine services. Nine (60%) through a secure Google Forms digital platform, and 6 (40%) using paper-based questionnaires. Paper forms were subsequently digitized and encoded into a centralized spreadsheet for consolidated analysis and evaluation.

Healthcare providers indicated a high-performance expectancy, appreciating telemedicine’s ability to enhance job performance by effectively managing patient health, especially in remote areas with limited access to specialty care. This is crucial for addressing complex medical conditions and improving healthcare quality in underserved regions. Effort expectancy was also rated positively, as telemedicine’s user-friendly interface made it easier for generalist providers to integrate this technology into their daily practices, thereby broadening the scope of care they can offer.

Despite neutral responses regarding social influence, which suggests minimal peer pressure to adopt the technology, the practical benefits of telemedicine emerge as the primary drivers of its adoption. Positive feedback on facilitating conditions suggests that adequate organizational and technical support significantly enhances the effective implementation and utilization of telemedicine services.

Additionally, hedonic motivation suggests that respondents find telemedicine enjoyable and engaging, which may lead to increased user engagement. Telemedicine’s cost-effectiveness, as reflected in its positive price-value perception, underscores its viability as a cost-effective solution for healthcare delivery.

The high behavioral intention to use telemedicine, driven by these positive perceptions, highlights its transformative potential in enhancing healthcare delivery within resource-limited settings. Telemedicine’s practical benefits further support this strong inclination to adopt it, making it a valuable tool in healthcare.

### Health system context

For the qualitative component, 20 participants took part in key informant interviews and focused group discussions, comprising medical officers, program implementers, and IT support staff from participating facilities.

#### Technical context

Implementing telemedicine in the Zamboanga Peninsula showcases substantial advancements in extending specialty medical services to remote and underserved areas. However, the efficacy of these services is often compromised by technical challenges such as limited internet connectivity, complex user interfaces, and inadequate hardware (See Table 1). These issues are particularly pronounced in emergency and inpatient departments, where the time required for data entry can be substantial, sometimes taking several hours to complete. This not only delays treatment but also strains the resources of the facilities involved. Enhancing the user experience, especially on mobile platforms, is crucial, as the existing desktop-optimized interfaces do not adequately meet users’ needs in areas with fluctuating connectivity.

**Table 1:** Selected quotes from participants about the factors affecting the utilization of the ZCMC telemedicine center

Factors	Themes	Quotes
Technical factors	Time consumption	‘*The problem is that it’s time-consuming, especially since we are on duty, and it’s complicated. It could take, for example, around 30 minutes per patient.*’ (Respondent 1) ‘*So far for me, doc, the first time I used it, ah... that thing, it... it consumed a lot of time, primarily when I handled OPD cases since my status is doc; more on OPD cases now, previously because I was ROD, but I used it when I was having my OPD, so it took me, I think, 5 hours to dispose of the patient.*’ (Respondent 2)
	Interface problems	‘*Yes, so I still have to ask the patient then, so there are things that I still need to ask the patient, like, I think, there are certain things I forgot what those are, but now it’s lesser though there are still things that I need to ask the patient and then when it comes to the case report, I think those need to be asked. However, there’s no option that if unavailable, you can just skip it.*’ (Respondent 1) ‘*But supposedly, it’s required to have one [a cellphone number], I no longer put it, or I just make up some data because there’s no way to skip, there are no options to skip or like no options to put N/A or unavailable or they don’t have a number.*’ (Respondent 1) ‘*The real challenge is the app’s interface because it’s for desktop, so what we have are cellphones; we can do referrals, but it’s tasking; it will take us one hour just for one patient.*’ (Respondent 3) ‘*Ah, first and foremost, doctor, the encoding is the issue because it’s not very user-friendly in terms of the format.*’ (Respondent 2)
	Internet connectivity	‘*That’s just how it is in the afternoon, ma’am, because it seems the internet is really slow, but it’s not always the same because sometimes in the morning it’s very slow, and I just keep trying... still nothing... the patients, you know, get discouraged, they go home and then come back late... sometimes I find out, I’m the only one answering back.*’ (Respondent 4)
	Hardware limitations	‘T*he computer hangs whenever we run the application.*’ (Respondent 5) ‘*The real challenge is the app’s interface because it’s for desktop, so what we have are cellphones; we can do referrals, but it’s tasking; it will take us one hour just for one patient.*’ (Respondent 13)
Organizational influences	Support and training	“*I [as the hospital chief] highly encourage them to use telemedicine since they are all GPs... Since all our GPs are here, I encourage them to refer their complex cases to the consultants... for telemedicine... since you are offering us this kind of help, I encourage them to do so. Still, I cannot force them to use the program because it’s convenient for them; they each have known consultants to whom they turn.*” (Respondent 2) ‘*She [referring to the telemedicine operator] learned on her own, which I’m impressed with because when she entered into telemedicine, she told me “ok, teach me,” but I couldn’t teach her yet because I was really busy at that time, I was seldom here, and eventually, she just learned on her own.*’ (Respondent 6) ‘*When the outgoing nurse told me one day that I’ll be taking over, I got to it immediately. I studied how to do it. It took me just about one day learning it, ma’am; it was just like texting.*’ (Respondent 14)
	Mixed views on workload management	‘*Yes, there are other doctors on duty, but they’re not... even though I’ve explained the process to them, it seems like they don’t want to because it seems like a burden to them (a burden to them, because of additional workload), additional workload (they have to enter data too), yes.*’ (Respondent 5) ‘*For me, it’s become easier because others, before we had to think about what to do, now we refer right away and get immediate answers, we don’t have to deal with the simple cases anymore, we treat them right here in jail without the need to take them outside right away. So it’s easier.*’ (Respondent 17)
	Available medicine and supplies	‘*I inform the consultant, “Doc, this is all we have available, can we just use this?” and they simply tell us that it won’t cover everything until it’s negative, if possible, have it purchased. And we just inform whether it’s been bought or not*.’ (Respondent 3) ‘*So far, all services [telemedicine] are okay, maybe just the availability of medicines if ever we can find a way around it.*’ (Respondent 8)
Political concerns	LGU support	‘*So that’s the difficulty because it seems that telemedicine is just allowed, but there’s also no support, like okay, just do it, it’s fine... something like that.*’ (Respondent 1) ‘*It seems like there’s no support from the LGU, doctor, because it’s still under the province, so all the resources needed, the province still has to lobby for them.*’ (Respondent 3) ‘*Ah, because the thing [coordination for the implementation of telemedicine] is that this was through the LGU, not through the hospital, so basically it’s the LGU itself that is providing support (the LGU itself), so whatever they can support, they give it, although the hospital itself is supportive in terms like for example when there are cases like that.*’ (Respondent 19)
Ethical concerns	System privacy and security	‘*Regarding the telemedicine policy at ZCMC... the data privacy policy. That’s just our part. It’s a bit new, and currently, we don’t see any problem with the platform where we transmit the data of patients as it is secure, because, eventually, we’re moving toward making telemedicine an integral part of the UHC.*’ (Respondent 6)
Legal influence	Lack of policy integration	“*Since all our GPs are here, I highly encourage them to refer their difficult cases to the consultants... to telemedicine... because you are offering us this kind of help, so I encourage them to do so, but I cannot force them to use the program because they have their convenience, they know their consultants to ask...unless maybe, doctor, if there is a mandate from the region to really (Yes) to use this... this platform for the proper referrals since there are no policies from the region, from the DOH, so I also don’t want to make a policy from the hospital because it might... tread on their rights*.” (Respondent 2)
Sociodemographic factors	Patient preference and accessibility	‘*Actually, doctor, it’s better now because the system is good since contacting the right doctor is easier. For example, if there are patients we advise to go to Zamboanga but don’t want to, they just bring them back home, for instance. So through telemedicine, we manage them here rather than have them travel to Zamboanga.*’ (Respondent 10) ‘*It’s okay, actually. They (Muslims, IPs, Subanen) really like it, it’s easier for them this way.*’ (Respondent 10)

#### Organizational factors

Organizational structures within healthcare facilities play a pivotal role in adopting and effectively utilizing telemedicine. Elements such as workload management, healthcare system practices, and the capacity to adapt to technological innovations are integral to this process. Facilities with strong support mechanisms report more substantial benefits from telemedicine, facilitating effective care management and continuous professional development for medical staff. However, the high staff turnover in rural areas and the lack of interoperability with existing hospital information systems present significant barriers, leading to inefficiencies and underutilization of the technology. Strategic organizational adjustments and ongoing training are crucial for addressing these challenges and enhancing service delivery.

#### Ethical concerns

Ethical considerations are paramount in deploying telemedicine, with a particular focus on the privacy and confidentiality of patient data. While telemedicine protocols generally include consent mechanisms, healthcare providers still notice a digital and cybersecurity literacy deficiency. This gap underscores the need for comprehensive training programs and the development of robust ethical guidelines to ensure patient information is handled securely and responsibly.

#### Political and legal influences

The political and legal landscape significantly influences the implementation and sustainability of Telemedicine Centers. Political actors’ lack of enthusiasm and the absence of clear, standardized guidelines impede telemedicine initiatives’ effective governance and scalability. Establishing a legal framework that supports telemedicine practices is critical to ensuring the integrity and effectiveness of these services. Such frameworks should facilitate the integration of telemedicine into existing health policies and regulatory structures, promoting broader adoption and standardization across healthcare settings.

#### Socioeconomic factors

Telemedicine has been particularly well-received among economically disadvantaged and remote populations, who benefit from the reduced need to travel for specialist consultations. Therefore, the economic and social implications of telemedicine extend beyond mere healthcare delivery, affecting broader accessibility and equity in health services. The technology alleviates the financial and logistical burdens associated with traditional healthcare models, thereby enhancing the overall quality of life for these populations.

## DISCUSSION

### Benefits of telemedicine

Access to specialty services has traditionally been challenging in rural areas due to limited healthcare infrastructure and a shortage of skilled healthcare workers [11, 12]. Telemedicine has emerged as a viable solution for providing specialist care to patients with complex cases in remote locations, significantly enhancing the availability of specialty healthcare in the Zamboanga Peninsula. This integration of telemedicine technology enables patients to receive high-quality care management from specialists, regardless of their geographic location, thereby reducing the need for long-distance travel and enhancing the efficiency of healthcare delivery, as seen in the study of Angelpolulou et al. (2022) [13].

Furthermore, the Telemedicine center represents a dual innovation in policy and technology that has transformed the healthcare landscape in the Zamboanga Peninsula, offering specialty medical consultations to remote and underserved populations. It has been effectively implemented across various healthcare settings, enhancing continuous learning for medical residents and nurses, and improving the efficiency and productivity of healthcare facilities in remote areas. The program’s benefits extend beyond improving the delivery, access, and efficiency of health systems during and after the COVID-19 pandemic, particularly for vulnerable populations [14].

Institutions such as prisons have also benefited from telemedicine, facilitating the efficient processing of court-order documents and ensuring timely medical care for persons deprived of liberty, thereby improving their overall health outcomes. Additionally, telemedicine has opened new possibilities for people in remote areas with limited access to specialized medical care. By enabling patients to receive medical consultations and treatments locally, telemedicine saves time and money while improving patient outcomes and quality of life [15].

### User acceptance and utilization

In the Zamboanga Peninsula, healthcare professionals demonstrate a strong inclination towards telemedicine, influenced by perceived performance expectancy, effort expectancy, facilitating conditions, hedonic motivation, and price value. Consistent with the study of Palma et al. in Brazil, the likelihood of telemedicine usage increases when professionals perceive institutional and governmental support through appropriate policies, guidelines, and the provision of necessary resources such as healthcare personnel, medicines, and medical supplies [16]. Additionally, their intent to use telemedicine is further shaped by their perceptions of how the technology enhances their work performance, the program’s efficiency, and ease of use, alongside the cost–benefit analysis for their facility and patients [17].

Technical challenges, including time-consuming data entry processes, complex user interfaces, unreliable network connectivity, and inadequate hardware, are significant barriers to the effective utilization of telemedicine in the Zamboanga Peninsula. These technical issues may impact the efficiency and performance of telemedicine services, influencing healthcare providers’ satisfaction and adoption rates. For example, interfaces that are not user-friendly can delay essential data entry, which is crucial for making quick medical decisions. At the same time, poor connectivity can disrupt vital virtual consultations, potentially compromising patient care.

Addressing these technical barriers is crucial, especially considering the individual factors such as performance and effort expectancy identified by healthcare professionals. These factors suggest that healthcare providers are more likely to utilize technology effectively when telemedicine systems are perceived as efficient and supportive of improved job performance. Therefore, enhancing system usability and interoperability can significantly boost their confidence and willingness to utilize telemedicine services [18].

Beyond technical aspects, continuous support and comprehensive training are crucial for addressing organizational influences. Ensuring that all personnel are proficient with the telemedicine center through ongoing training, particularly, in provinces with high staff turnover, is essential for maintaining service standards. Moreover, effective workload management also aligns with healthcare workers’ hedonic motivation by reducing burnout and increasing job satisfaction, encouraging the sustained use of telemedicine. The availability of medical supplies and local government support also play essential roles. These elements ensure that telemedicine serves as a consultation channel and facilitates care management, which is necessary in remote areas where handling complex cases is challenging.

Optimizing technical configurations to improve user experience and reliability, and addressing facilitating conditions through a system-wide support system, can significantly enhance the potential and effectiveness of telemedicine [19, 20]. These improvements will help realize the benefits identified by healthcare professionals in their performance expectancy, effort expectancy, and behavioral intention for telemedicine, leading to better healthcare outcomes across the Zamboanga Peninsula.

### Implications to policy and practice

Despite its benefits, implementing telemedicine faces several organizational and technical challenges. Inadequate training, high staff turnover, and the need for improved interoperability with existing hospital systems are prevalent issues that complicate the integration of telemedicine into routine practice. Moreover, the legal and ethical frameworks governing telemedicine remain underdeveloped, raising concerns about data privacy and the management of patient information.

The findings suggest a pressing need for the development of comprehensive regional policies to govern the integration of telemedicine within primary healthcare services [19]. Such policies should facilitate the allocation of necessary resources and support the standardization of practices across healthcare settings. Enhancing telemedicine infrastructure to include more user-friendly features, such as video communication capabilities, could significantly improve the efficacy of medical consultations and decision-making processes [21, 22].

To maximize its impact, telemedicine should be integrated into a comprehensive health system framework that encompasses supportive policies, a skilled workforce, and accessible medical resources. Continuous training and orientation for healthcare personnel are essential to ensure the proficient use of telemedicine technologies. This approach will address current implementation challenges and enhance the scalability and sustainability of telemedicine services, potentially leading to universal health coverage in the region [23–25].

### Limitations of the study

In the region, it is common for healthcare professionals, particularly physicians, to be employed on a non-regular basis. This employment instability often leads to high turnover rates and scheduling uncertainties among healthcare personnel, complicating staffing continuity across various healthcare facilities. These challenges posed significant difficulties in constructing a comprehensive sampling frame for users of the telemedicine center in rural areas. Consequently, the study employed purposive sampling, selecting participants based on specific criteria, such as their availability and active use of telemedicine in their respective facilities, rather than using random sampling methods.

Additionally, the limited number of respondents in the UTAUT 2 survey constrained the study’s statistical power. To manage this limitation, the research employed descriptive statistics, including means and contingency tables, which were calculated using Spearman’s Rho test. This approach was necessary to summarize and elucidate the relationships between individual factors and the behavioral intentions of telemedicine users. Despite these methodological constraints, the study provided valuable insights into the factors influencing the adoption and use of telemedicine among healthcare professionals in the region.

## CONCLUSION

This case study examined the implementation of the RTC established by a tertiary medical center in Southwestern Mindanao, with a focus on its operational structure, digital infrastructure, and integration within the local health system. The RTC operated under a centralized governance structure led by the apex hospital, with formal agreements and structured workflows enabling provider-to-provider referrals. Its web-based platform allowed frontline healthcare workers to consult specialists despite geographic and resource constraints. As of 2024, the RTC enabled remote specialist consultations across ‘34 partner facilities’, utilizing a custom-developed web-based platform to facilitate structured specialist referrals, case triage, and asynchronous communication between providers. This system-wide referral counts totals ‘1793 cases as of June 2024’.

Quantitative findings from the pilot survey, based on the UTAUT2, revealed that healthcare personnel perceived the RTC platform as both valuable and easy to use, reflected in high scores for performance expectancy and effort expectancy. Positive responses for hedonic motivation and price value further suggest satisfaction and perceived cost-effectiveness, contributing to a high behavioral intention to integrate telemedicine into routine workflows.

Qualitative findings from focus group discussions and key informant interviews deepened the analysis by identifying influencing adoption. Common ‘technical barriers’ included intermittent connectivity, hardware limitations, and occasional usability issues. Organizational challenges, including high staff turnover, inadequate retraining, and limited system interoperability, were identified as disrupting continuity and consistency. ‘Ethical and legal concerns’, particularly relating to data privacy and consent, remained unresolved in the absence of clear institutional or national frameworks. Furthermore, ‘political and policy-related constraints,’ such as variable local government support and a lack of regulatory integration, shaped program scalability. ‘Socioeconomic realities,’ including patient expectations, geographic remoteness, and cultural norms, also contributed to variation in utilization across sites.

Taken together, these findings affirm that while ‘individual-level acceptance’ of the RTC is promising, its ‘sustainable and scalable implementation’ requires structural and systemic enablers. The RTC demonstrates the feasibility of a digitally enabled, regionally coordinated model of specialist referral for underserved areas. However, its long-term impact depends on continuous investment in ‘infrastructure, human resource development, data governance,’ and ‘policy alignment’. By documenting the program architecture and field-level experiences, this case study contributes actionable insights for embedding telemedicine into a resilient, equity-driven, and learning health system, particularly in resource-constrained settings.

## Data Availability

Both the quantitative and qualitative data underlying this article will be shared upon reasonable request to the corresponding author.
